# Between the Borders: Balint Syndrome as a Rare Manifestation of Posterior Circulation Stroke

**DOI:** 10.7759/cureus.97550

**Published:** 2025-11-23

**Authors:** Bushra Khan, Ashna Arif, Fathalla Elnagi

**Affiliations:** 1 Department of General Medicine, Whiston Hospital, Prescot, GBR; 2 Department of Internal Medicine, Whiston Hospital, Prescot, GBR; 3 Department of Stroke, Whiston Hospital, Mersey and West Lancashire Teaching Hospitals NHS Trust, Prescot, GBR

**Keywords:** balint syndrome, optic ataxia, posterior circulation stroke, simultagnosia, watershed infarct

## Abstract

Balint syndrome is a rare disorder caused by bilateral parieto-occipital damage, most often occurring within the posterior watershed territory between the middle and posterior cerebral arteries. It is characterized by a triad of simultanagnosia, optic ataxia, and oculomotor apraxia, and is often misdiagnosed as delirium, dementia, or cortical blindness, particularly in elderly patients. We report a case of an 82-year-old woman who presented with confusion and visuospatial disorientation following a collapse. Her visual acuity was preserved, but she demonstrated simultanagnosia and oculomotor apraxia. Computed tomography of the brain revealed bilateral occipital watershed infarcts, consistent with Balint syndrome secondary to posterior border-zone infarction. She was managed with secondary stroke prevention and commenced on neurorehabilitation focusing on visuospatial and visuomotor retraining. This case highlights the importance of recognizing Balint syndrome as a rare manifestation of posterior circulation stroke. Awareness of its classic triad facilitates early diagnosis, appropriate neuroimaging correlation, and tailored rehabilitation to optimize patient outcomes.

## Introduction

Border-zone or watershed infarcts occur in regions of the brain located between the major arterial territories. These strokes can affect both cortical and subcortical areas. Cortical border-zone strokes most commonly occur between the middle cerebral artery (MCA) and posterior cerebral artery (PCA) in the posterior region, and between the anterior cerebral artery (ACA) and MCA in the anterior region. These territories are vulnerable to hypoperfusion and embolic-watershed mechanisms [1]. The clinical presentation depends on both the anatomical location and whether the infarct extends into adjacent cortical or white matter regions.

In the posterior watershed region (MCA-PCA interface), bilateral infarction may present as a characteristic triad of simultanagnosia (the inability to perceive more than one object at a time), optic ataxia (misreaching for visual targets), and oculomotor apraxia (difficulty in voluntary gaze shifting); collectively known as Balint syndrome [2]. Conversely, infarction in the anterior watershed region (ACA-MCA interface) can result in “man-in-the-barrel” syndrome, characterized by proximal upper limb weakness with preservation of distal and lower limb function [3].

Due to the rarity of these presentations, they are often under-recognized in acute stroke settings. We report a case of bilateral posterior watershed infarction presenting as Balint syndrome, highlighting the importance of clinical recognition and contrasting it with the anterior watershed presentation of man-in-the-barrel syndrome.

## Case presentation

An 82-year-old woman presented to the emergency department following an episode of confusion and collapse. Her past medical history included hypertension and three previous strokes. On examination, her visual acuity was preserved; however, she exhibited marked visuospatial disorientation. She was unable to perceive multiple objects simultaneously, consistent with simultanagnosia. In addition, she demonstrated oculomotor apraxia, requiring head movements to compensate for difficulty in shifting gaze towards visual targets. Lastly, optic ataxia was evident on examination as the patient consistently misreached for objects presented in the visual field. However, visual field testing demonstrated no clinical evidence of neglect.

**Figure 1:**
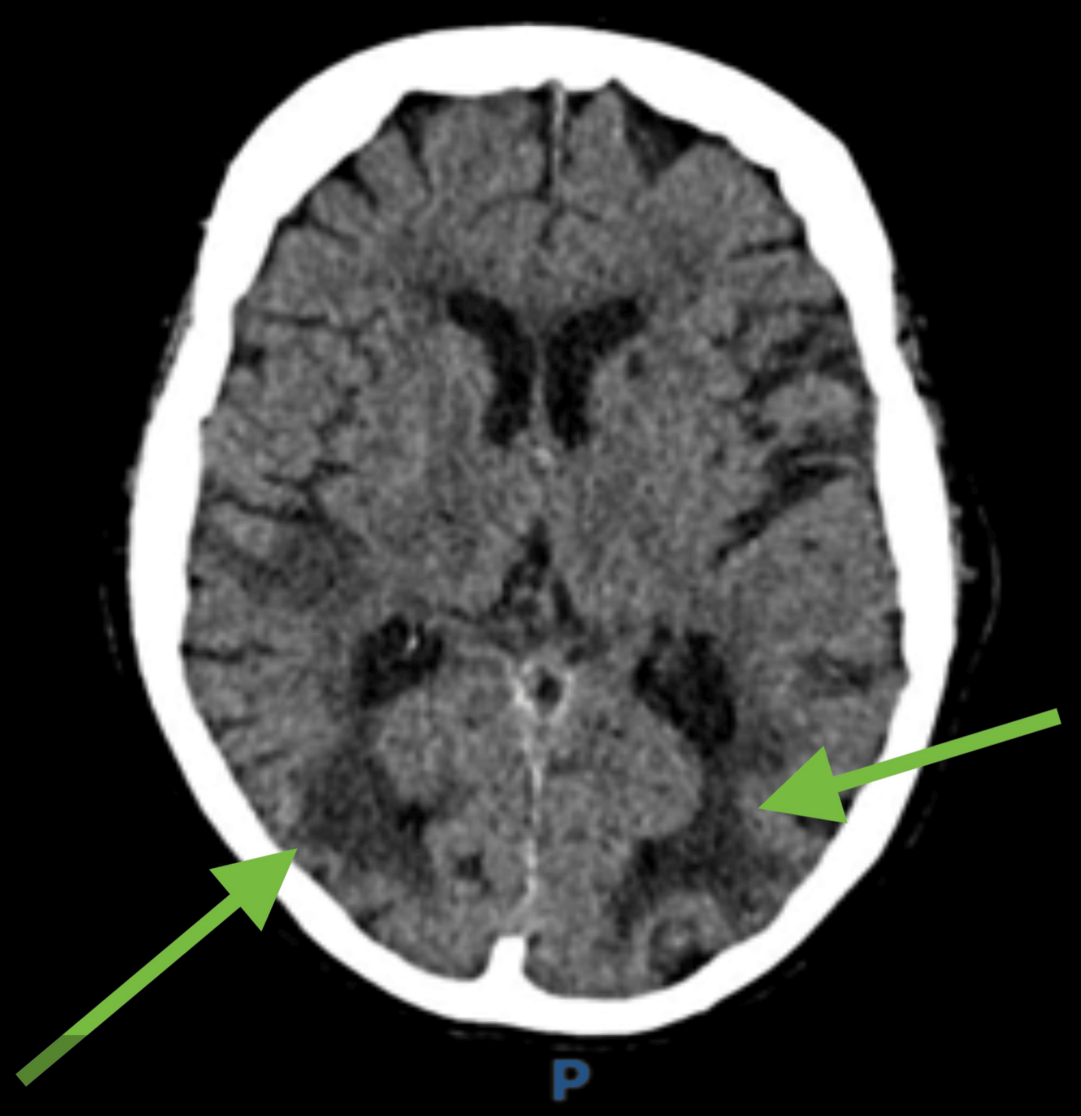
Non-contrast CT brain imaging revealed bilateral occipital and posterior parietal hypodensities (green arrow).

Sensory examination was preserved, and there were no attention or memory deficits. Motor strength was intact, and there were no speech abnormalities or focal motor deficits. Non-contrast CT brain imaging revealed bilateral occipital and posterior parietal hypodensities, predominantly affecting the cortical grey matter; consistent with watershed infarcts (Figure 1).

Based on the triad of simultanagnosia, oculomotor apraxia, and optic ataxia, a diagnosis of Balint syndrome secondary to bilateral watershed infarction was made. She was admitted to the stroke unit, commenced on secondary prevention therapy, and referred for neurorehabilitation focusing on visuospatial and visuomotor retraining.

## Discussion

Posterior circulation strokes account for approximately 20-25% of all strokes in the United Kingdom [4]. Balint syndrome, as a rare manifestation of posterior watershed infarction, can be easily overlooked in the acute setting. These features are often misinterpreted as delirium, dementia, or cortical blindness, particularly in elderly patients, leading to delayed recognition [5,6]. Prompt identification of this syndrome enables targeted therapy and optimized rehabilitation, ultimately improving patient outcomes [7].

In contrast, the anterior watershed syndrome, or “man-in-the-barrel” syndrome, arises from bilateral anterior cerebral artery (ACA)-MCA infarcts. It typically presents with proximal upper-limb weakness while sparing hand and lower-limb strength, giving the impression of being “trapped in a barrel” [8].

Table 1 summarizes the key differences between Balint syndrome and man-in-the-barrel syndrome. As discussed above, both syndromes can arise from bilateral watershed infarcts, and the corresponding signs reflect their location [9,10]. This comparison highlights the regional specificity of watershed injury, with this patient demonstrating posterior parieto-occipital territory involvement consistent with Balint syndrome.

**Table 1:** Comparison between Balint syndrome and man-in-the-barrel syndrome. MCA: middle cerebral artery; PCA: posterior cerebral artery

Features	Balint syndrome	Man-in-the-barrel syndrome
Vascular territory	Posterior border zone (MCA-PCA)	Anterior border zone (MCA-ACA)
Core deficits	Visuomotor disintegration: optic ataxia, oculomotor apraxia, simultanagnosia	Proximal upper limb weakness with preserved distal/lower limb strength
Lesion site	Bilateral parieto-occipital cortex	Bilateral frontoparietal white matter/centrum semiovale
Functional impact	Higher-order visual-spatial processing	Motor execution (proximal arms)

CT brain imaging demonstrated lesions localized to the parieto-occipital cortices, which are particularly vulnerable to hypoperfusion, explaining the selective cortical injury observed. However, an MRI would have better shown the characterization of lesion depth and white matter involvement. Although quantitative infarct analysis was beyond the scope of this report, future cases could benefit from volumetric or diffusion-weighted assessment to better define structure-function relationships.

This patient underwent an early transfer to a neurorehabilitation unit and exhibited substantial improvement in functional abilities, such as visual attention and hand-eye coordination. This highlights the importance of facilitating timely rehabilitation to enhance functional outcomes in these patients.

## Conclusions

Balint syndrome is a rare but clinically important manifestation of bilateral posterior watershed infarction. Awareness of the classic triad - simultanagnosia, oculomotor apraxia, and optic ataxia - is essential, particularly in elderly patients presenting with visuospatial disorientation and preserved visual acuity. Early recognition facilitates correlation with neuroimaging, reduces the risk of misdiagnosis, and enables timely, targeted rehabilitation. Comparison with anterior watershed presentations, such as man-in-the-barrel syndrome, underscores the importance of understanding these syndromes within the broader spectrum of stroke pathophysiology.
